# Sparse logistic regression revealed the associations between HBV PreS quasispecies and hepatocellular carcinoma

**DOI:** 10.1186/s12985-022-01836-9

**Published:** 2022-06-28

**Authors:** Jian-an Jia, Shuqin Zhang, Xin Bai, Meng Fang, Shipeng Chen, Xiaotao Liang, Shanfeng Zhu, Danny Ka-Ho Wong, Anye Zhang, Jianfeng Feng, Fengzhu Sun, Chunfang Gao

**Affiliations:** 1 Department of Laboratory Medicine, Eastern Hepatobiliary Surgery Hospital, Navy Military Medical University, Shanghai, 200438 China; 2 Department of Laboratory Medicine, The 901th Hospital of Joint Logistics Support Force of Chinese People’s Liberation Army, Hefei, 230031 China; 3 Centre for Computational Systems Biology, School of Mathematical Sciences, Fudan University, Shanghai, 200433 China; 4 Molecular and Computational Program, Department of Biological Sciences, University of Southern California, 1050 Childs Way, Los Angeles, 90089 USA; 5 Department of Computer Science, Fudan University, Shanghai, 200433 China; 6 Institute of Science and Technology for Brain-Inspired Intelligence, Fudan University, Shanghai, 200433 China; 7 State Key Laboratory for Liver Research, The University of Hong Kong, Hong Kong SAR, China; 8 Department of Medicine, The Eighth Affiliated Hospital, Sun Yat-Sen University, Shenzhen, 518033 China; 9 Department of Computer Science, University of Warwick, Coventry, CV4 7AL UK; 10 Clinical Laboratory Medicine Center, Yueyang Hospital of Integrated Traditional Chinese and Western Medicine, Shanghai University of Traditional Chinese Medicine, Shanghai, 200437 China

**Keywords:** Hepatocellular carcinoma, HBV quasispecies, Next generation sequencing (NGS), Sparse logistic regression (SLR), PreS region

## Abstract

**Background:**

Chronic infection with hepatitis B virus (HBV) has been proved highly associated with the development of hepatocellular carcinoma (HCC).

**Aims:**

The purpose of the study is to investigate the association between HBV preS region quasispecies and HCC development, as well as to develop HCC diagnosis model using HBV preS region quasispecies.

**Methods:**

A total of 104 chronic hepatitis B (CHB) patients and 117 HBV-related HCC patients were enrolled. HBV preS region was sequenced using next generation sequencing (NGS) and the nucleotide entropy was calculated for quasispecies evaluation. Sparse logistic regression (SLR) was used to predict HCC development and prediction performances were evaluated using receiver operating characteristic curves.

**Results:**

Entropy of HBV preS1, preS2 regions and several nucleotide points showed significant divergence between CHB and HCC patients. Using SLR, the classification of HCC/CHB groups achieved a mean area under the receiver operating characteristic curve (AUC) of 0.883 in the training data and 0.795 in the test data. The prediction model was also validated by a completely independent dataset from Hong Kong. The 10 selected nucleotide positions showed significantly different entropy between CHB and HCC patients. The HBV quasispecies also classified three clinical parameters, including HBeAg, HBVDNA, and Alkaline phosphatase (ALP) with the AUC value greater than 0.6 in the test data.

**Conclusions:**

Using NGS and SLR, the association between HBV preS region nucleotide entropy and HCC development was validated in our study and this could promote the understanding of HCC progression mechanism.

**Supplementary Information:**

The online version contains supplementary material available at 10.1186/s12985-022-01836-9.

## Introduction

Hepatocellular carcinoma (HCC) is the most common type of primary liver cancer. Individuals with chronic HBV infection are at increased risk of developing HCC, especially among those with chronic liver disease and cirrhosis [1–3].

HBV population presents in the form of quasispecies consisting of a large number of genetically heterologous variants in the host [4]. As the reverse transcriptase in HBV replication lacks proofreading activity, the HBV replication error rate is much higher than other DNA virus. Thus, various mutations can be observed in the HBV quasispecies during long-term infection [5]. Some mutations can serve as potential viral markers for predicting the development and progression of HBV-associated HCC. HBV integration sites, HBV genotypes, basal core promoter mutations, precore mutations, and preS deletions have all been implicated in the development of HCC [5–16].

The double-stranded DNA (dsDNA) genome of HBV contains four overlapping open reading frames. The preS region (nucleotides 2854-155) consisting of preS1 and preS2 fragments overlaps a region of the polymerase gene. Selection and emergence of naturally occurring, or therapeutically induced, HBV variants with mutations in the preS/S genomic region are frequent events in chronically HBV-infected patients. In particular, specific mutations in the preS/S region may induce an unbalanced production of envelope proteins that accumulate in the endoplasmic reticulum (ER) of the hepatocytes, potentially activating ER stress-signaling pathways with consequent induction of oxidative DNA damage and genomic instability [17]. Thus, the preS region may have more clinical implications for the development of HCC.

The development of next-generation sequencing (NGS) technologies has greatly accelerated genome studies. NGS can simultaneously sequence a large number of viral quasispecies with high sensitivity and specificity [18] and NGS has been widely implemented in the exploration of HBV low-frequency drug resistance [19, 20]. Comparative studies between NGS and the previous sequencing techniques in HBV studies have shown the advantages of NGS [21, 22]. Several works have been done on the associations between preS mutations and HCC using NGS in recent years [16, 23–25]. In the researches [16, 23], the authors mainly applied statistical test to analyze the associations between preS mutations and HCC. Our team [25] also studied the associations between preS deletions and HCC, and used Support Vector Machine (SVM) to check the prediction results of the identified associated preS deletions. Our team also applied word patterns of HBV genome to define the distance between HCC patients, and the heterogeneity of HBV genotypes and the associations between word patterns of HBV preS region and HCC [25]. Other investigators also have reported the predictive effect of preS deletions [6, 7, 11, 26–28] in HCC development. While the association between HBV preS quasispecies with HCC has been probed in limited studies [16, 29], it should be investigated more deeply.

In this work, we investigated the associations between HCC and HBV quasispecies based on NGS of the preS region. The quasispecies in preS region of chronic hepatitis B (CHB) and HCC patients were explored. With state-of-the-art statistical learning methods such as sparse logistic regression (SLR), we built a prediction model of HCC/CHB with HBV quasispecies. The positions that contribute to the associations were also analyzed. The associations between the HBV quasispecies and the clinical parameters were studied as well.

## Materials and methods

### Source of data and participants

This study follows the Transparent Reporting of a multivariable prediction model for Individual Prognosis or Diagnosis (TRIPOD) report [30] (Additional file 5: Table S1). HCC patients were enrolled between March 2011 and May 2012 at the Eastern Hepatobiliary Surgery Hospital, Shanghai, China. HBV-related HCC patients fulfilled following criteria: (1) serum hepatitis B virus surface antigen (HBsAg) positive at least 6 months; (2) HBV DNA levels > 1000 IU/ml; (3) HCC characteristic confirmed by operative findings and histopathological examination. The exclusion criteria included hepatitis C virus or human immunodeficiency virus co-infection, a history of liver transplantation, autoimmune liver diseases, metastatic liver cancer, other malignancies, drug-related liver diseases, alcoholic hepatitis and other causes of chronic liver diseases diagnosed before enrollment. CHB patients included fulfilled criteria including: (1) serum HBsAg positive at least 6 months; (2) continuous or repeatedly serum alanine aminotransferase (ALT) elevation (two times above the upper reference range for no other reason than HBV infection) or chronic viral hepatitis characteristic confirmed by liver biopsy; (3) HBV DNA levels > 1000 IU/ml. The exclusion criteria included HCC, the malignancies or other serious disease. This study was approved by The Ethics Committee of the Eastern Hepatobiliary Hospital (EHBHKY2015-01–004). Serum samples were collected from all patients before hepatectomy. Totally, 104 CHB samples and 117 HCC samples were amplified and sequenced successfully, with 63 CHB patients (CHB group) and 46 HBV-related HCC patients (HCC group) in the training set (Shanghai dataset), and 41 CHB and 71 HCC samples in the test set (Shanghai dataset). For the HCC patients, we also collected their clinical examination data.

### HBV DNA extraction and Illumina sequencing in preS region

HBV genomes were extracted from 200 μl of serum samples using the QIAamp DNA Mini kit (QIAGEN GmbH, Hilden, Germany) and eluted in 100 μl of distilled water. The preS region was amplified using Phanta Super-Fidelity DNA Polymerase (Vazyme Biotech, Piscataway, New Jersey, USA) with a pair of primers: 5′-CGCCTCATTYTKYGGGTCA-3′ (forward, nucleotides 2801–2819), and 5′-TCCKGAACTGGAGCCACC-3′ (reverse, nucleotides 62 to 79). PCR amplicons of the preS region were purified with Agencourt AMPure XP beads (Beckman Coulter, Beverly, Massachusetts) and were quantified with the Qubit dsDNA HS assay kit (Invitrogen, Carlsbad, CA, USA). A library of PCR products of the preS region was prepared using the TruSeq DNA PCR-Free sample preparation kit (Illumina, San Diego, CA, USA) and was run on a MiSeq sequencer (Illumina, San Diego, CA, USA) for paired-end sequencing, according to Illumina protocol. Finally, fluorescent signals were analyzed using the MiSeq control software and transferred to sequence data in the FASTQ format.

### Sequence read mapping and genotyping

Quality evaluation of raw reads was performed with the online tool fastqc (http:// www.bioinformatics.babraham.ac.uk/projects/fastqc/), and the reads having average base calling quality score under 20 were discarded. After quality filtration and adapter removal, paired-end reads were joined with FLASH, v1.2.10 [31]. Merged preS region sequence was genotyped with HBV STAR software as reported previously [32], and corresponding preS regions of 23 reference HBV genomes from the GenBank database were used for genotyping (Accession numbers: X02763, X51970, AF090842, D00329, AB073846, AB602818, X04615, AY123041, AB014381, X65259, M32138, X85254, X75657, AB032431, X69798, AB036910, AF223965, AF160501, AB064310, AF405706, AY090454, AY090457, AY090460). The genotype of each sample was defined as the most frequent one among all 8 types from A to H.

### External validation

This dataset includes 32 HBV-related HCC patients and 32 CHB patients without HCC (Hong Kong dataset) and patients were enrolled between July 2007 and December 2012 in the Hepatitis and Liver Clinic, Queen Mary Hospital, University of Hong Kong, Hong Kong [16]. Serum samples were collected and sequenced. More details about patients enrollment and HBV sequencing can be found in [16]. Except the Illumina MiSeq platform used in deep sequencing, all the other platforms and tools are different from what we used when generating our data. We got the data from the researchers [16], and used BLAST to map merged reads (fasta format) into HBV reference genome. According to the mapping results, reads with insertions, deletions and turnovers were filtered out. If the normal reads percentage of a sample is less than 20, we removed the sample. Finally, we obtained the data for 26 HCC and 23 CHB patients. The sequence includes 589 nucleotide acids, of which 457 ones are overlapped with the fragment sequenced in our study. We only considered the same 457 positions as those in our dataset for this dataset.

### Data preprocessing and predictors

After sequencing the quasispecies, we collected the point mutation data for 457 positions including the positions from 1 to 61 and 2820 to 3215 in and close to the preS region. We counted the frequencies of the nucleotides in each position. To describe the mutation complexity in each position, we transformed the frequency data to Shannon entropy, which is defined as $H = - \sum\nolimits_{i} {p_{i} } \log p_{i}$, $\sum\nolimits_{i} {p_{i} } = 1$ where $i \in \{ A,C,G,T\}$ and *p*_*i*_ is its frequency, $x\log (x) = 0$ when *x* = 0. Entropy of all the 457 nucleotide positions of preS region were used as predictors for HCC diagnosis.

### Model development and validation

We applied Sparse Logistic Regression (SLR) to model the associations between HCC/CHB groups and quasispecies. SLR is to add the term $\lambda \left\| {\beta_{1} } \right\|$ to the original logistic regression model, where $\beta$ is the coefficient vector of the variables. This model can simultaneously conduct classification and variable selection. By tuning the parameter $\lambda$, we can obtain the sparse form of $\beta$ with the nonzero entries corresponding to the selected variables. The independent variables in our study include the entropy data of the 457 positions, and the response variables denote patients belonging to the CHB or HCC group. We aim to model the associations between the 457 positions and the CHB/HCC group. We applied *K*-fold cross-validation (CV) to select the parameter $\lambda$ such that $\beta$ is the sparsest among those achieving accuracy within one SD of the highest accuracy. Then we applied the fitted model using all training data with selected $\lambda$ to the test set to see the prediction performance. We directly implemented the function: glmnet() in the R package ‘glmnet’ [16] by setting alpha = 1, which is a parameter to balance the contributions between $\left\| {\beta_{1} } \right\|$ and $\left\| {\beta_{2} } \right\|$. With alpha being 1, the $\left\| {\beta_{2} } \right\|$ term will not contribute to the model, and less variables will be selected with the same classification accuracy. We used four criteria to evaluate the performance of the model in our experiments: accuracy, area under the ROC curve (AUC), sensitivity, and specificity.

### Association between the clinical parameters of HCC patients and quasispecies

For the categorical clinical parameters and those quantitative parameters following non-normal distributions, we applied SLR, as above described. For the parameters following normal distribution, we applied Sparse Partial Least Square regression (SPLS), a method designed to find the combination of all independent variables so as to be most correlated with the response variable. Here, we also imposed *l*_1_ penalty to obtain a sparse solution of the coefficients. We adopted the method proposed in [34] and directly used the R package ‘spls’ [34]. To choose the number of latent components (combinations) κ and the soft threshold η to determine the zero entries of the coefficients, we also used CV to tune the parameters. We first fixed η and varied κ to choose the best κ and then fixed κ to choose the best η.

## Results

In this section, we presented our main findings on the relations between nucleotide point entropy in preS region and HCC development. Our main aim is to classify the CHB/HCC patients or build the prediction model for HCC using nucleotide point entropy in the preS region, at the same time, to find some important point mutations that contribute to HCC development. Since clinical indexes are more easily obtained, we also explored the associations between point mutations in preS region and clinical indexes.

### Participants and nucleotide acid entropy of the preS region

The baseline information of the CHB and HCC patients (Shanghai dataset) was summarized in Table 1. In both training and test cohort, the HCC patients showed more inferior liver function, older age and lower serum HBV DNA levels.

**Table 1:** Demographics and baseline laboratory markers of training cohort and test cohort

	Training cohort		Test cohort	
	CHB (n = 63)	HCC (n = 46)	CHB (n = 41)	HCC (n = 71)
*Gender*				
Female	21 (33.3%)	6 (13.6%)	18 (43.9%)	13 (18.3%)
Male	42 (66.7%)	38 (86.4%)	22 (53.7%)	58 (81.7%)
Age	36.6 ± 13.8	52.4 ± 8.3	40.1 ± 11.9	51.1 ± 10.2
Serum HBV DNA	7.48	5.02	6.24	5.32
(log_10_ IU/ml)	(6.50–7.83)	(4.43–6.25)	(4.89–7.65)	(4.80–5.85)
TBIL (μmol/L)	13	14.2	11	14.5
	(10.0–17.0)	(11.1–19.8)	(8.7–15.7)	(11.5–19.4)
DBIL (μmol/L)	3	5.5	4	5.7
	(3.0–5.0)	(4.0–7.7)	(3.0–6.0)	(4.4–7.5)
TP (g/L)	74	68.8	75	67.8
	(71.5–79.0)	(64.4–71.1)	(70.0–78.0)	(64.1–72.7)
ALB (g/L)	45.5	39.8	45	40.9
	(41.0–49.7)	(37.0–42.5)	(43.0–48.5)	(38.0–43.9)
ALT (U/L)	38	43.4	51	40.5
	(19.0–66.0)	(24.7–78.4)	(29.5–77.0)	(33.0–71.0)
AST (U/L)	30	46	33	40
	(21.0–45.0)	(25.0–84.5)	(26.5–54.2)	(31.0–64.0)
GGT (U/L)	22	81	24	78
	(15.0–51.0)	(41.3–156.0)	(18.0–43.2)	(50.0–121.5)
ALP (U/L)	71.5	92	72	92
	(59.299.7)	(69.25–131.5)	(56.0–82.5)	(73.5–112.0)
*HBsAb*				
Negative	17 (40.5%)	36 (85.7%)	38 (97.4%)	64 (95.5%)
Postive	25 (59.5%)	6 (14.3%)	1 (2.6%)	3 (4.5%)
*HBeAg*				
Negative	31 (73.8%)	25 (59.5%)	8 (20.5%)	41 (61.2%)
Postive	11 (26.2%)	17 (40.5%)	31 (79.5%)	26 (38.8%)
*HBeAb*				
Negative	33 (78.6%)	10 (23.8%)	31 (79.5%)	18 (26.9%)
Postive	9 (21.4%)	32 (76.2%)	8 (20.5%)	49 (73.1%)
*HBV genotype*				
B	26(41.2%)	8(17.4%)	15(36.6%)	12(16.9%)
C	37(58.7%)	36(78.3%)	26(63.4%)	59(83.1%)
Tumor size(cm)		5.6(3.32–9.40)		11(7.8–14.5)
*Capsule*				
Intact		5 (11.4%)		10 (15.6%)
None		5 (11.4%)		11 (17.2%)
Partial		34 (77.3%)		43 (67.2%)
*Tumor number*				
1		32 (72.7%)		58 (90.6%)
2		0 (0.0%)		3 (4.7%)
3		12 (27.3%)		3 (4.7%)
*PVTT*				
None		23 (52.3%)		45(63.3%)
Yes		21 (47.7%)		19 (29.7%)
AFP(ng/ml)		565 (29.1–15,375.0)		187 (7.83–1210.0)

*AFP* Alpha-fetoprotein, *ALB* Albumin, *ALP* Alkaline phosphatase, *ALT* Alanine aminotransferase *AST* Aspartate aminotransferase *CHB* Chronic hepatitis B, *DBIL* Direct bilirubin, *GGT* γ-glutamyltransferase; *HBV* Hepatitis B virus, *HBeAg* Hepatitis B e antigen, *HBeAb* Hepatitis B e antibody, *HBsAb* Hepatitis B antibody, *HCC* Hepatocellular carcinoma, *TBIL* Total bilirubin, *TP* Total protein, *PVTT* Portal veint umor thrombus

**Fig. 1:**
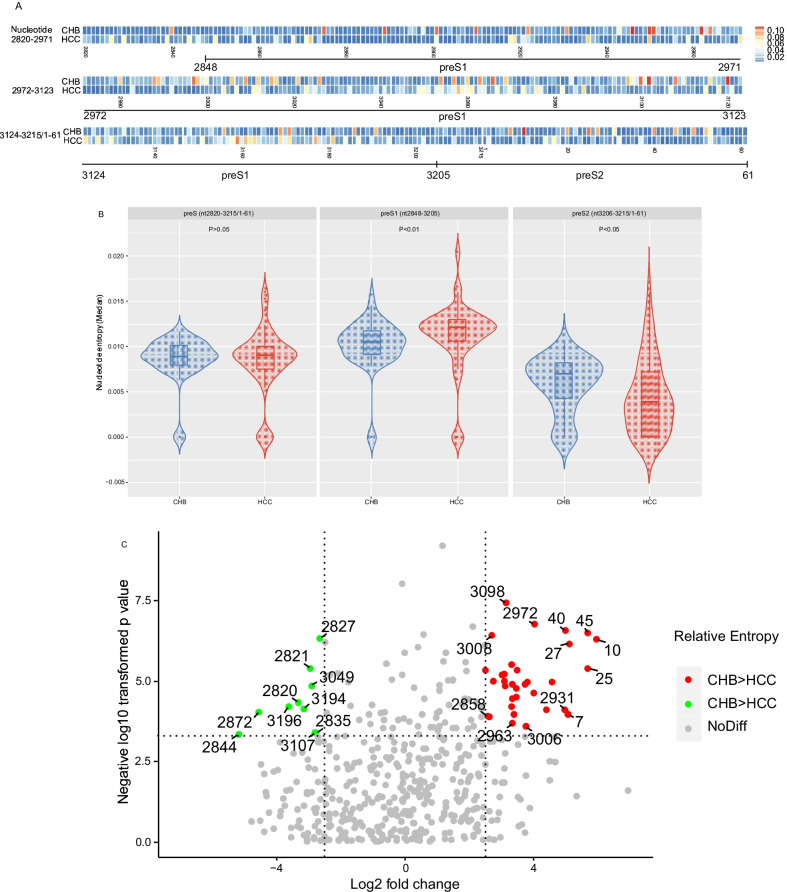
HBV preS region nucleotide entropy of CHB and HCC patients. **A**. Heat map show the nucleotide entropy in preS region of CHB and HCC patients. **B**. Comparison of nucleotide entropy in preS, preS1 and preS2 region between in CHB and HCC patients. **C**. Nucleotide points in preS region with different entropy between in CHB and HCC patients were described by volcano plot filtering. Entropy of all the nucleotide points in preS region were compared between in CHB and HCC patients. After logarithm, the *p*-values were presented in the y-lab direction. In the x-lab direction, the relative entropy ratios of CHB and HCC patients were also log-transformed and presented. Red spots represented nucleotide points with higher entropy in CHB patients, of which *p*-values and fold changes were upon specific threshhold. The green spots mean nucleotide points with opposite conditions

The nucleotide acid entropy of preS region was calculated and the entropy distribution was shown in Fig. 1A. The median entropy of preS region in CHB patients was 0.0087 (0.0074–0.0092), which is lower than counterpart in HCC patients 0.0090 (0.0076–0.01001). No significant difference was found between entropy of all nucleotide points in preS region (Fig. 1B). When nucleotide points entropy of the preS1 and preS2 were compared respectively, nucleotide points entropy in preS1 region of HCC patients were significantly higher than those in CHB patients. While in preS2 region, the opposite trend was presented between HCC and CHB patients (Fig. 1B). Furthermore, entropy of individual nucleotide positions was compared and the *p*-value and fold-changes were presented in Fig. 1C. A lot of positions showed significant divergence in entropy between CHB and HCC patients.

### Model development and performance

**Fig. 2:**
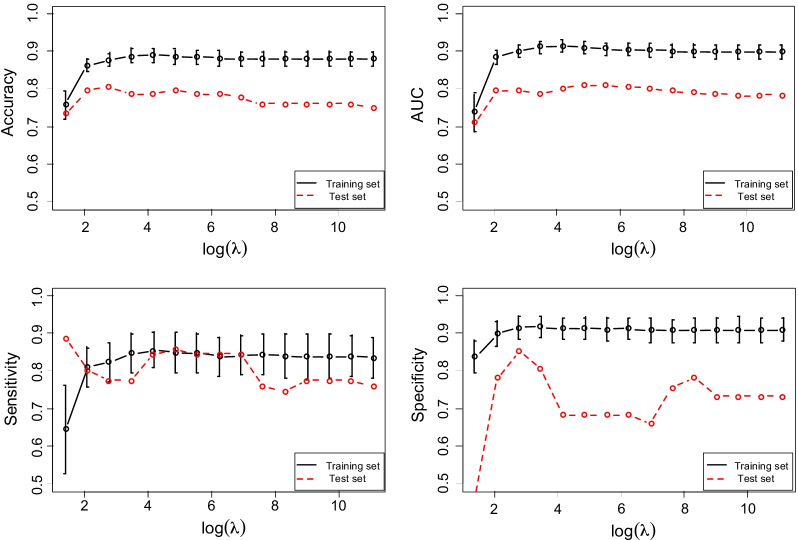
Classification results for HCC/CHB patients using SLR with different parameters. Classification results (accuracy, AUC, sensitivity and specificity) for all HCC/CHB patients as a function of penalization parameter λ in sparse logistic regression. The vertical lines show one SD in the CV studies

Since too many nucleotide positions with divergent entropy exist between CHB and HCC patients, more sophisticated methods should be applied to investigate the associations between nucleotide entropy and HCC development. Thus, we studied the classification of HCC/CHB groups with quasispecies data using SLR [33]. The model was fitted with the training dataset (46 HCC/63 CHB, Shanghai dataset), and was applied to do the prediction in the test sets (71 HCC/41 CHB, Shanghai dataset). To tune the parameter λ that controls the selection of the variables (nucleotide positions), we ran fivefold CV 50 times in the training set. The value of λ started from 0.5^2^ with a proportion of 0.5 to decrease, and the length of λ was set as 15. Figure 2 shows the prediction results for all λ’s. In the training data, when λ is less than 0.5^3^ (the 2nd point), the four evaluation criteria are all stable, with sensitivity having the greatest SD. In the test set, both accuracy and AUC were stable starting from λ = 0.5^3^. Here, λ was chosen as 0.5^3^, and Table 2 shows the classification results. The accuracy and AUC achieved a mean value of 0.861 (SD = 0.032) and 0.883 (SD = 0.043) in the training set and 0.794 and 0.795, respectively, in the test set. The SLR model performed more superior than classic logistic regression model in Table 2. This shows the high associations between HBV quasispecies and HCC development.

**Table 2:** Classification results for HCC/CHB using LR, SLR, SVM and SSVM

	LR	SLR (λ = 0.5^3^)		SVM	SSVM (λ = 0.5^5^)
	Test set	Training set	Test set	Test set	Test set
Accuracy	0.688	0.861 (0.032)	0.794	0.777	0.679
Sensitivity	0.718	0.809 (0.051)	0.803	0.775	0.662
Specificity	0.634	0.898 (0.019)	0.780	0.780	0.707
AUC	0.644	0.883 (0.043)	0.795	0.836	0.685

*LR*: We applied the trained model in training set to the test set (Test set). *SLR*: We did cross validation within the training set (Training set), and applied the trained model to the test set (Test set). *SVM*: We applied the trained model in training set to the test set (Test set). *SSVM*: We did cross validation within the training set, and applied the trained model to the test set (Test set)

*AUC* Area under the receiver operating characteristic curve; *CHB* Chronic hepatitis B; *HCC* Hepatocellular carcinoma; *LR* Logistic regression; *SLR* Sparse logistic regression; *SVM* Support vector machine; *SSVM* Sparse support vector machine

The final obtained prediction model using SLR is:

$$\begin{aligned} \log \left( {\frac{p}{1 - p}} \right) = & - 0.793 - 0.670x_{10} - 0.224x_{45} \\ & \quad + 1.169x_{2858} + 0.157x_{2861} + 0.046x_{2992} + 1.381x_{3046} \\& \quad + 1.125x_{3090} + 0.824x_{3093} + 1.487x_{3098} + 0.235x_{3207} \\ \end{aligned}$$

where the subscript of each variable means the point mutation positions that were selected. For each sample, after the entropy of each position is calculated, the sample is centralized by subtracting the mean entropy. Then the above formula is applied to compute the probability of being CHB or HCC, with a smaller probability leading to CHB.

Other machine learning methods were also investigated. We compared the above results with those obtained using Support Vector Machine (SVM) [35, 36] and Sparse Support Vector Machine (SSVM) [37]. SVM is a popular classification method in machine learning, which classifies the samples using all the considered variables. Similar to SLR, SSVM is formulated as a hinge loss function with an *l*_1_ penalty term to select the associated variables when doing classification [37]. We implemented SVM using the R package ‘e1071’, and SSVM using R package ‘sparseSVM’ [37], respectively. Using similar procedure as SLR, we trained the model using the training set and applied it to the test set. The prediction results in the test dataset are also shown in Table 2. For SSVM, we also did model calibration using the R package ‘platt’ [38], which implements Platt calibration. Platt calibration is to transform the classification outputs into a probability distribution over classes by fitting a logistic regression model to a classifier’s scores. The performance of SSVM can be improved after calibration. Since SLR outputs the probability for each sample being HCC patient, we directly gave its calibration plot. The prediction results and the reliability diagrams of both SLR and calibrated SSVM were put in Additional file 1: Figure S1 and Additional file 6: Table S2, which shows similar performance. Though the AUC for SVM is higher than that of SLR, it cannot identify the associated variables. The performance of both SSVM and calibrated SSVM is much worse than SLR. Thus, our following analysis for CHB/HCC classification is based on SLR.

#### Independent validation in the Hong Kong dataset

We first applied SLR to the entropy data of the Hong Kong dataset as the training set. Owing to the small sample size, we used tenfold CV 50 times to conduct the experiments within this dataset and record the results. The value of λ was finally chosen to be 0.5^6^. Table 3 shows the results in ‘Training results’ (Hong Kong dataset). Within the Hong Kong data, mean accuracy and AUC achieved a value of 0.822 (SD = 0.031) and 0.724 (SD = 0.054), respectively. We then used the model trained by Shanghai dataset to predict the HCC/CHB patients in the Hong Kong dataset as the test set. The results are shown in ‘Test results’ in Table 3. The prediction has accuracy 0.694 and AUC 0.607, respectively. The independent sequencing experiments further confirmed HCC development is associated with HBV quasispecies.

**Table 3:** Classification results for HCC/CHB from Hong Kong dataset using SLR

	Training results	Test results
Accuracy	0.822 (0.031)	0.694
Sensitivity	0.717 (0.071)	0.500
Specificity	0.931 (0.053)	0.913
AUC	0.724 (0.054)	0.607

Training results: we did cross validation within the Hong Kong dataset using SLR with λ = 0.5^6^. Test results: we applied the model trained in Shanghai dataset using sparse logistic regression and took Hong Kong dataset as the test set

*AUC* Area under the receiver operating characteristic curve; *CHB* Chronic hepatitis B; *HCC* Hepatocellular carcinoma; *SLR* Sparse logistic regression

#### Nucleotide position quasispecies associated with HCC development

**Fig. 3:**
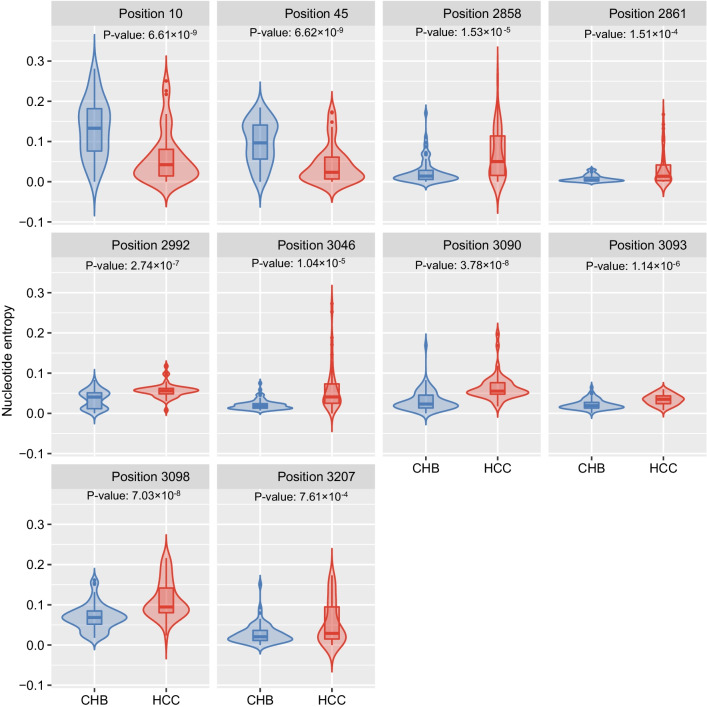
Comparison of entropy of the ten nucleotide points of HBV preS region selected by SLR model. Ten nucleotide points of HBV preS region were selected by SLR model for CHB/HCC classification and entropy between in CHB and HCC patients were compared. All of the ten nucleotide points showed significant divergence between in CHB and HCC patients

We checked the coefficientsin the model trained with our whole training data when λ = 0.5^3^. Ten positions were selected to be associated with HCC: 10, 45, 2858, 2861, 2992, 3046, 3090, 3093, 3098, and 3207. When λ became less than 0.5^3^, except position 45, the remaining 9 positions were kept in the model. This shows the high associations between HCC and the 9 nucleotide positions. In Fig. 3, the entropy of these nucleotide points were significantly different between HCC and CHB patients. We carried out *t*-test for the entropy data of these 10 positions. All of them were significantly different between HCC and CHB patients with a minimum *p*-value in position 45 and a maximum *p*-value in position 3207. The base frequencies of these 10 positions in HCC and CHB patients were also presented in Additional file 2: Figure S2, respectively and it is clear that the base distributions are also different in HCC and CHB samples.

#### Effect of training sample size on the prediction accuracy of HCC/CHB patients

**Fig. 4:**
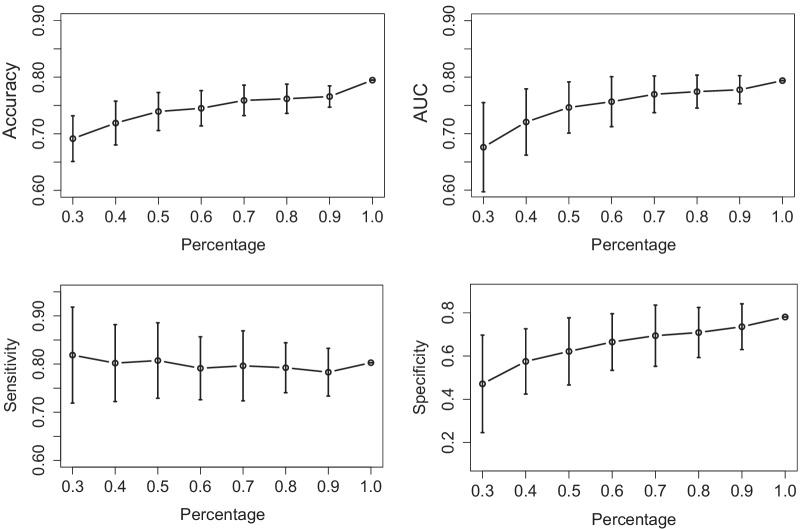
Classification results for HCC/CHB patients using SLR with different sample size. The vertical lines show one SD in the cross validation studies

Since with limited samples, the models developed may have potential to perform worse when applied to new patients, we further did experiments to evaluate the predictive models and to see how the performance of SLR depends on the sample size [39]. We randomly selected a given percentage of samples from the original training set with λ = 0.5^3^ to train the model, and then applied it to the test data. The percentage varied from 30 to 100 percent, with 100 percent corresponding to all the training samples. We repeated samplings for each percentage 50 times. The mean for all four evaluation criteria and the SD were plotted in Fig. 4. When the sample size increased, the accuracy, AUC and specificity all correspondingly increased, while SD decreased. Sensitivity was relatively stable and had a value around 0.80. Even with only 30 percent of the data, that is, only 33 training samples, the mean accuracy and AUC were around 0.70 and 0.67, which are higher than that obtained using ordinary LR and comparable to that using SSVM. When using 70 percent of the training samples, the number of which is less than 80, the results were much better than that of LR and SSVM, and became stable. These show the efficiency of the SLR model in our experimental settings.

### Genotype analysis of all patients

To see the differences between the samples of different genotypes, we studied the samples of genotype C and genotype B separately. With the same model training method, we chose λ = 0.5^8^ and λ = 0.5^3^ for patients of genotype B and genotype C, respectively. The results for different λ’s are shown in Additional file 3: Figure S3, and the results for the chosen λ are listed in Table 4. Compared to results that using all the patients, the specificity increased and the sensitivity decreased for patients of genotype B. Meanwhile, the specificity decreased and the sensitivity increased by several fold for patients of genotype C.

**Table 4:** Classification results for HCC/CHB patients of different HBV genotypes using SLR

	Genotype B (λ = 0.5^8^)		Genotype C	Genotype C (λ = 0.5^3^)		Genotype B
	Training set	Test set*	Test set^#^	Training set	Test set*	Test set^#^
Accuracy	0.924(0.028)	0.778	0.741	0.870 (0.023)	0.776	0.741
Sensitivity	0.678(0.122)	0.583	0.915	0.840 (0.064)	0.864	0.500
Specificity	0.999(0.006)	0.933	0.346	0.890 (0.036)	0.577	0.933
AUC	0.861(0.061)	0.706	0.754	0.843 (0.027)	0.767	0.722

*Test results were produced using SLR model trained by patients with the same genotype of HBV

^#^Test results were produced using SLR model trained by patients with the other genotype of HBV

*AUC* Area under the receiver operating characteristic curve; *CHB* Chronic hepatitis B; *HCC* Hepatocellular carcinoma; *SLR* Sparse logistic regression

We also performed cross-prediction as a check on prediction performance. We trained the model with genotype C or B patients in the training set and predicted the other genotype patients in the test set. The results were added in Table 4. Accuracy and AUC were both comparable to those within the same genotype, while sensitivity and specificity showed more changes. Thus, for prediction purposes, this finding implies that we might combine all individuals together to produce a larger sample size, as demonstrated by our experiments.

### Association between HBV quasispecies and clinical parameters in HCC patients

For the HCC patients, we investigated the associations between HBV quasispecies and clinical parameters. For the categorical clinical parameters and those quantitative parameters following non-normal distributions, we applied SLR. For the parameters following normal distribution, we applied SPLS [39]. Owing to the small sample size, we ran tenfold CV 50 times to choose the parameters λ, η, and κ. When we applied the SPLS model, η was set between 0.1 and 0.9 with a step size of 0.1, and κ varied between 2 and 10. If the AUC for the independent test was greater than 0.60, we took the clinical parameter as being associated with HBV quasispecies. Finally, we found that the serum indexes: hepatits B e antigen (HBeAg), HBVDNA, and alkaline phosphatase (ALP) were associated with HBV quasispecies. The classification results for different values of λ and η were showed in Additional file 4: Figure S4. Table 5 shows the classification results for the selected λ and η.

**Table 5:** Classification results for clinical parameters using SLR in HCC patients

	HBeAg		HBV DNA		ALP	
	*λ* = 0.57		*κ* = 4, *η* = 0.8		*κ* = 2, *η* = 0.7	
	Training set	Test set	Training set	Test set	Training set	Test set
Accuracy	0.882 (0.026)	0.672	0.791(0.038)	0.676	0.782 (0.037)	0.634
Sensitivity	0.929 (0.042)	0.805	0.757(0.084)	0.667	0.712 (0.082)	0.703
Specificity	0.920 (0.056)	0.462	0.833(0.074)	0.690	0.868 (0.058)	0.559
AUC	0.882 (0.051)	0.602	0.697(0.052)	0.675	0.688 (0.048)	0.648

*AUC* Area under the receiver operating characteristic curve; *HCC* Hepatocellular carcinoma; *SLR* Sparse logistic regression; *ALP* Alkaline phosphatase; *HBV* Hepatitis B virus; *HBeAg* Hepatitis B e antigen

When classifying the HBeAg-positive and -negative patients in the training set, both accuracy and AUC were around 0.9. While the accuracy and AUC in the test set were 0.672 and 0.607, respectively. For the parameter HBVDNA, the accuracy and AUC were around 0.7 for all η’s in the training set. In the test set, the accuracy and AUC decreased to 0.676 and 0.675, respectively. Similarly, for ALP, both accuracy and AUC were stable with all η's around 0.8 and 0.7 in the training set. While the accuracy and AUC were 0.634 and 0.648 in the test set.

## Discussion

In this work, we investigated the quasispecies of HBV preS region in CHB and HCC patients using NGS method. No significant divergence was found in nucleotide entropy level of preS region between in CHB and HCC patients, which is not consistent with the previous study in [16]. Even so, nucleotide points’ entropy of preS region in HCC patient in higher in this study, which showed the same tendency as previous study [16]. Furthermore, obvious entropy divergence was observed in nucleotide entropy level of preS1 and preS2 respectively between the two patient groups. The patient group composition may contribute to the different results between the former study and ours. On the other hand, different fragments in genome may show unique nucleotide entropy and present special function.

Then we studied the associations between HCC and HBV quasispecies by applying SLR to the deep sequencing data of the preS region. The classification of HCC and CHB patients using entropy of the nucleotide frequency achieved a prediction accuracy of 0.794 and AUC of 0.795 in the independent test set, which are superior to the classic HCC marker: AFP [40, 41]. In another independent dataset from Hong Kong, the prediction accuracy and AUC were 0.695 and 0.607, respectively. These results demonstrate the high associations between HCC and HBV quasispecies. The decreasing of the accuracy and AUC in the Hong Kong dataset could be attributed to the different sequencing protocols, especially the different sequencing start and end points of the preS region. Since the target sequence was analyzed using large-scale parallel sequencing, even the minor divergence would be amplified thousands of times. Other reasons may include different sequencing process, different patients’ constitution, different intervention for the patients, and so on. The reason for this is worth further studying.

**Fig. 5:**
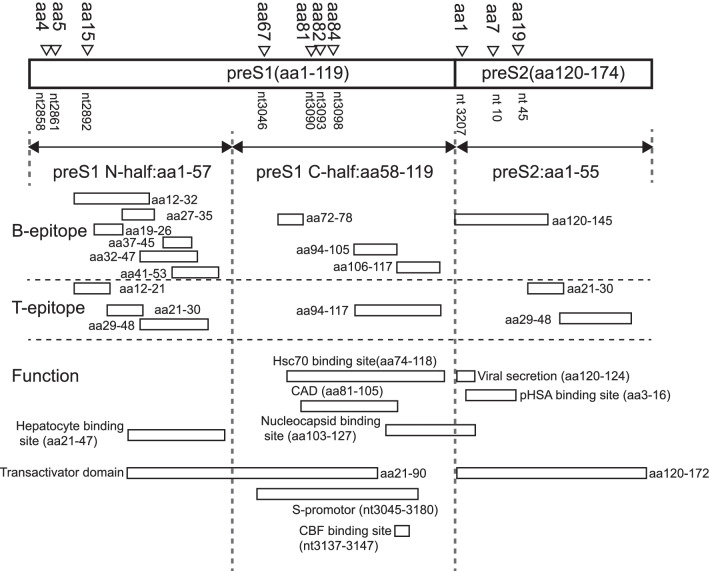
Nucleotide and amino acid mapping for the identified mutation points. Functions of ten nucleotide points of HBV preS region selected by SLR model for CHB/HCC classification are also denoted

One of the advantages of SLR is that significant variables could be selected accompanying the process of prediction model fitting. The 10 positions selected by SLR associated with HCC were significantly different in both entropy and nucleotide frequency data. We mapped the nucleotide of the selected positions to amino acid and checked the functional domains of these positions [6]. Figure 5 shows the nucleotide positions, corresponding amino acid and the functions. Except the position 2858 and 2861, all the remaining positions have related functions. Positions 3090, 3093, 3098, 3207, 10, 45 belong to the B cell epitope. Position 2892 belongs to the T cell epitope. Mutations in these epitopes may contribute to immune escape and affect virus-host immune interaction. Position 3046, 3090, 3093, 3098, 3207, 10, 45 are in the transactivator domain. Positions 3046, 3090, 3093, and 3098 locate in S-promoters region. These mutations may play roles in the process of virus gene transcription and expression. Position 10 belongs to the polymerized human serum albumin (pHSA) binding site which is involved in the process of virus binding and entry to hepatocyte and mutations in this region may have influence on virus-cell interaction [42]. Positions 3090, 3093 and 3098 all belong to the heat shock cognate 70 (Hsc70) binding site.

Among all 10 positions, three, including 10, 2858, and 3098, have been reported as being associated with HCC in the literature [16, 40–42]. The mutation C10A was reported as a risk factor for HCC patients compared to HCC-free HBsAg-positive patients [43]. Our study shows that the mutation in position 10 is significantly different between the HCC and CHB groups with *p*-value of 6.61 × 10^–8^. Besides the high mutation to ‘A’ reported in [43], we also found a high mutation to ‘T’. The mutation to ‘A’ is higher in the HCC group, while the mutation to ‘T’ is higher in the CHB group. Position 2858 was recently found to be associated with HCC development [16]. In this study, three codons, including 4, 27, and 167, were found to be associated with HCC development. Position 2858 belongs to codon 4. The mutation to ‘C’ of this position is much higher in HCC compared to CHB, as shown in Additional file 2: Figure S2. The mutation of T3098C is also showed association with HCC progress [43, 44] and the mutation ‘T’ to ‘C’ is much higher in HCC patients than in CHB patients. This is consistent with our study, as clearly shown in Additional file 2: Figure S2. Besides the mutation to ‘C’, we also found that the mutation to ‘G’ in HCC is higher than that in CHB in position 3098.

In the experiments for patients of genotypes B and C separately, the prediction performance was a little worse than that for all patients, but it still achieved accuracy and AUC greater than 0.7 in the independent test set. The prediction AUC decrease may be due to two reasons. Firstly, each genotype of virus may own its special classification features of disease status and these features may take effect in single genotype infection or mixed genotype infection. When patients infected with virus genotype mixture were separated by major infection genotype, features of the minor infection genotype could not take effect in disease status classification. Previous studies have shown the co-infection of different HBV genotypes is not unusual.[45, 46] So the classification efficiency decreased when patients were first grouped by infection genotype. Secondly, HCC progression was related with genotype C HBV infection [46, 47] and most HCC patients were infected with genotype C virus compared with CHB patients in our study (Chi-square test, *p*-value = 0.02). So some features that determine the infection genotype may also determine disease status. When the population was separated by infection genotype beforehead, these features could not further be used for disease classification, which also contributed to the decline of prediction effect.

The associations between the clinical examination parameters and the quasispecies for HCC patients were also studied. Three parameters, including HBeAg, HBVDNA and ALP, were found to be associated with HBV quasispecies. Previous studies have shown HBeAg and HBVDNA to be associated with HBV quasispecies [9, 49]. Our study further shows their associations in the preS region. ALP has not been reported by others and is, therefore, worth further study. All of the 3 parameters showed decreased AUC and accuracy in the test set compared to those in the training set. This could be attributed to the unbalanced data size of the training and test sets. We applied permutation test to check whether the associations between these three parameters and HBV quasispecies are artifactual. We permuted the response labels, and used the same method to see the prediction AUC. Then the AUCs from permuted data are significantly smaller than the AUC obtained from the real data (*p*-value < 0.05). This validates the existence of these associations. The selected positions associated with these three parameters are listed in Additional file 7: Table S3.

In our study, the main statistical methods are SLR and SPLS. With sparsity constraints, we can simultaneously find the associated variables and fit the prediction model. These methods have been applied to the study of disease-related biomarkers and classifications [34, 50, 51]. Though our team also used other machine learning methods and achieved better prediction efficiency based on HBV reverse transcriptase quasispecies [41], the SLR still has advantages, especially in variables selection, which could promote the understanding of prediction model as well as HCC progression mechanism.

In this study, we only focused on the point mutations though high associations between HCC and HBV quasispecies was showed. Other variations such as deletions, insertions also exist in the HBV genomes, and these nucleotide changes may have some linkages in the quasispecies variants. We did not take into account these issues in our current study. If these factors can be taken into account, better results should be expected. This is left as one of our future works. Furthermore, the limited sample size is a weakness of this study. It is better to estimate the sample size in advance according to criteria described in previous studies [52, 53], and use sufficient samples for predictive model development and validation. Finally, the degradation in performance of the external validation also implied that application of prediction model based on NGS should be strictly limited with preset conditions consisting of same sequencing strategy and bioinformatics analysis process.

## Conclusions

In this paper, using SLR, we proved the associations between HCC and HBV quasispecies of the preS regions probed by NGS. We also found ten point mutations in the preS region are associated with HCC development. Using these point mutations, the prediction of HCC/CHB patients shows promising results. These results help understanding the molecular mechanism driving the progression from HBV to HCC.

## Supplementary Information

**Additional file 1. Figure S1:** Reliability diagram for SLR without calibration (**A**), and calibrated SSVM using platt calibration (**B**). The vertical axis shows the proportion of the observed HCC patients in the dataset while the horizontal axis shows the predicted proportion of HCC patients. Since SLR outputs the probability for each sample being HCC patient, we directly give the calibration plot. We further calibrated SSVM using ‘platt’ and drew the plot. The results of SLR are similar to that of SSVM after calibration.

**Additional file 2. Figure S2:** Nucleotide base frequency in HCC and CHB individuals of the ten point mutation positions selected by sparse logistic regression.

**Additional file 3. Figure S3:** Classification results for HCC/CHB patients using SLR in patients with different genotype of HBV.**A** Classification results for HCC/CHB patients of genotype B for different λs. **B**. Classification results for HCC/CHB patients of genotype C for different parameter λs.

**Additional file 4. Figure S4:** Association between three clinical variables and HBV quasispecies displayed by SLR with different parameters. **A**. Association between HBeAg and HBV quasispecies for different λs. **B**. Association between HBVDNA and HBV quasispecies for different ηs when K = 4. **C**. Association between ALP and HBV quasispecies for different ηs when K = 2.

**Additional file 5. Table S1:** TRIPOD checklist.

**Additional file 6. Table S2:** Classification results for HCC/CHB using LR, SLR, SVM and SSVM and calibrated SSVM.

**Additional file 7. Table S3:** The selected positions associated with the three clinical parameters: HBeAg, HBVDNA, and ALP.

## Data Availability

The datasets generated and analyzed during the current study are available from the corresponding author on reasonable request.

## Abbreviations

HBV: Hepatitis B virus
CHB: Chronic hepatitis B
HCC: Hepatocellular carcinoma
LR: Logistic regression
NGS: Next generation sequencing
ROC Curve: Receiver operating characteristic curve
SLR: Sparse logistic regression
SPLS: Sparse partial least square
SVM: Support vector machine
SSVM: Sparse support vector machine
AFP: Alpha-fetoprotein
TBIL: Total bilirubin
DBIL: Direct bilirubin
TP: Total protein
ALB: Albumin
ALP: Alkaline phosphatase
ALT: Alanine aminotransferase
AST: Aspartate aminotransferase
GGT: γ-Glutamyltransferase
HBeAg: Hepatitis B e antigen
HBsAb: Hepatitis B antibody
HBeAb: Hepatitis B e antibody
PVTT: Portal veint umor thrombus
