# Supplementary material for: Sparse logistic regression revealed the associations between HBV PreS quasispecies and hepatocellular carcinoma
Source: Virol J. 2022 Jun 28;19:114. doi: 10.1186/s12985-022-01836-9 (PMC9238101; doi:10.1186/s12985-022-01836-9)
Supplement: Supplementary file 1 — Additional file 1. Figure S1: Reliability diagram for SLR without calibration (A), and calibrated SSVM using platt calibration (B). The vertical axis shows the proportion of the observed HCC patients in the dataset while the horizontal axis shows the predicted proportion of HCC patients. Since SLR outputs the probability for each sample being HCC patient, we directly give the calibration plot. We further calibrated SSVM using ‘platt’ and drew the plot. The results of SLR are similar to that of SSVM after calibration. [file 12985_2022_1836_MOESM1_ESM.pdf]

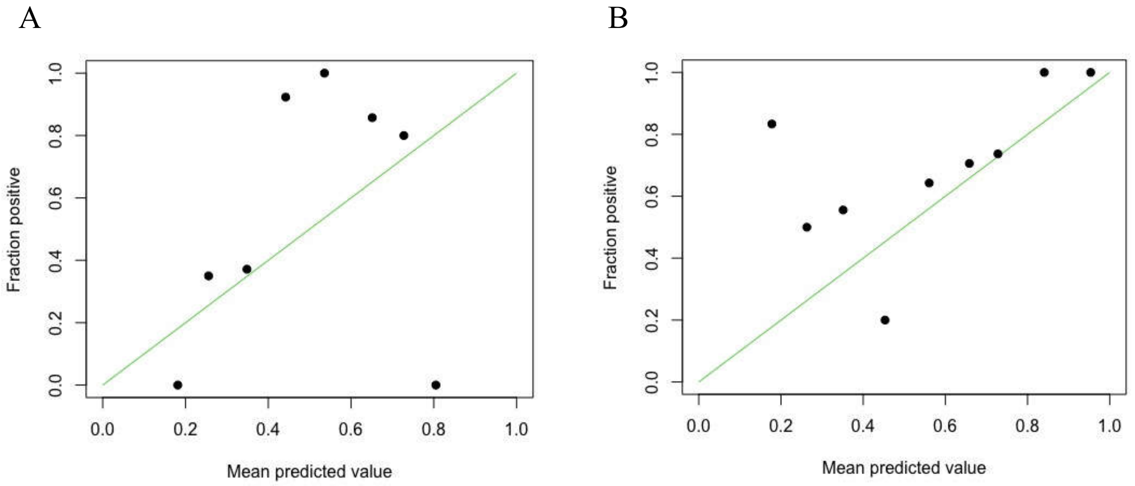

Figure S1. : Reliability diagram for SLR without calibration (A), and calibrated SSVM using platt calibration (B).
