# Supplementary figures and images for: Sparse logistic regression revealed the associations between HBV PreS quasispecies and hepatocellular carcinoma
Source: Virol J. 2022 Jun 28;19:114. doi: 10.1186/s12985-022-01836-9 (PMC9238101; doi:10.1186/s12985-022-01836-9)

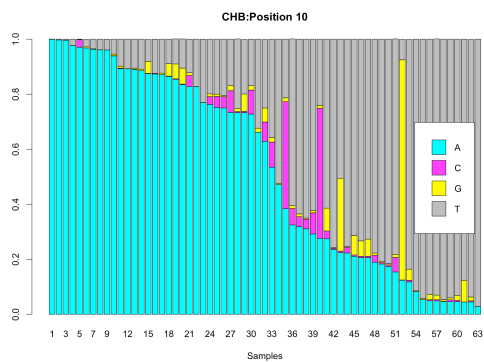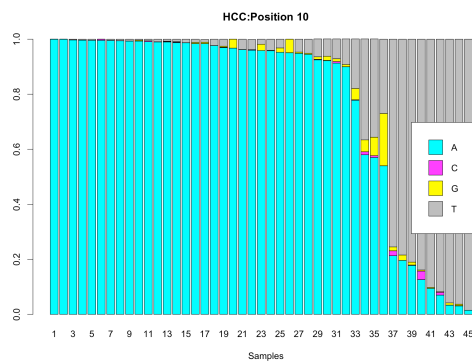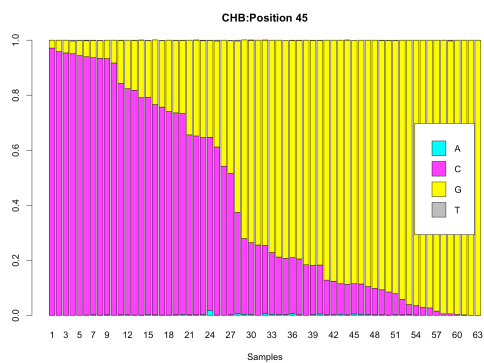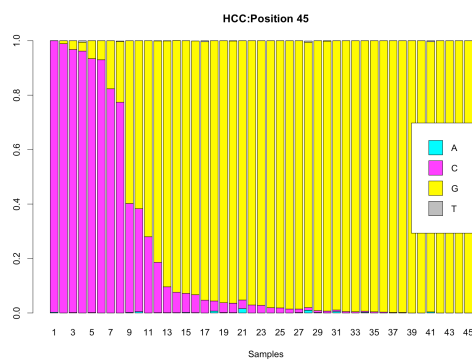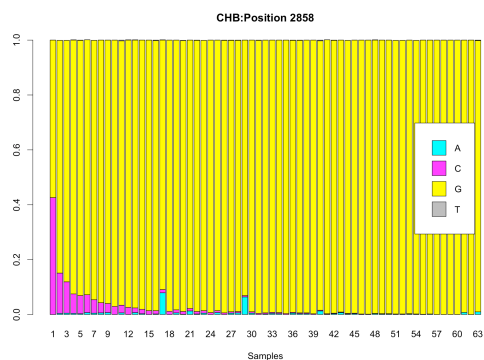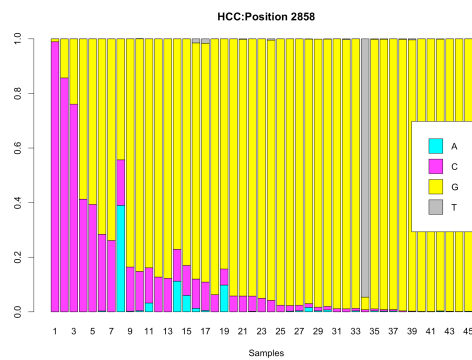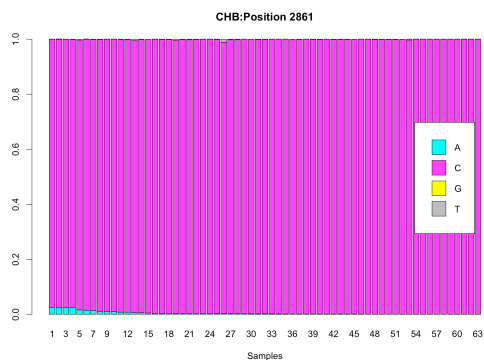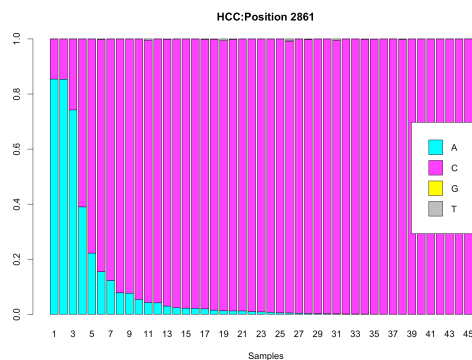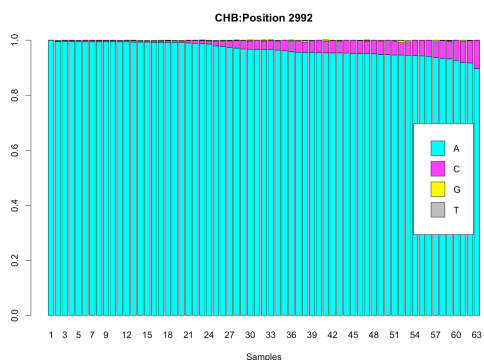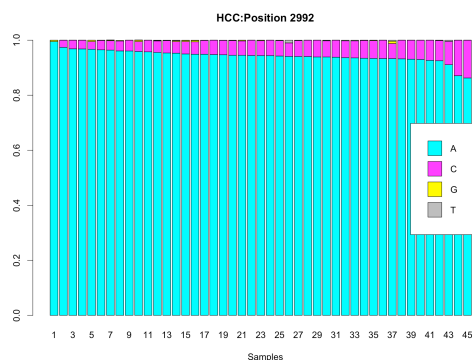

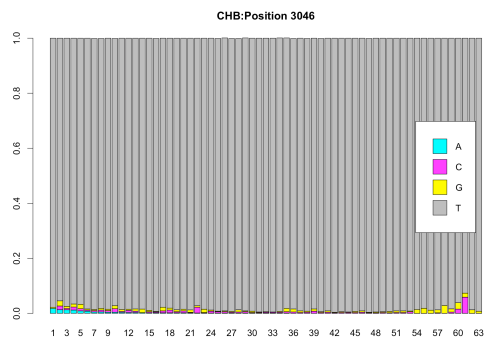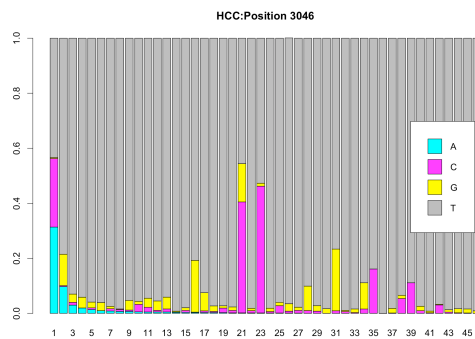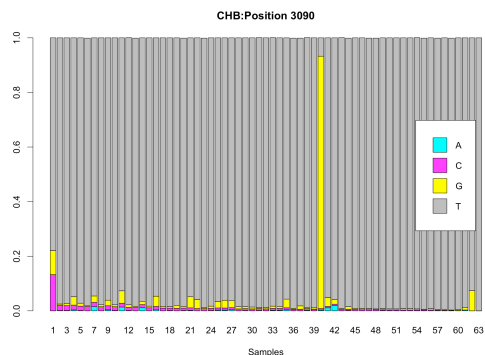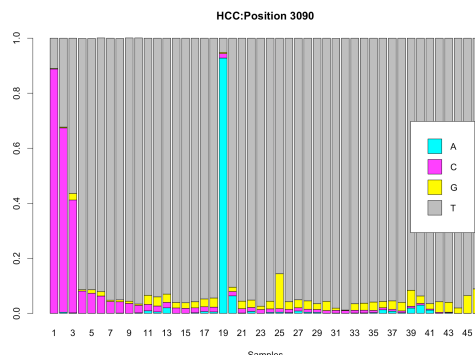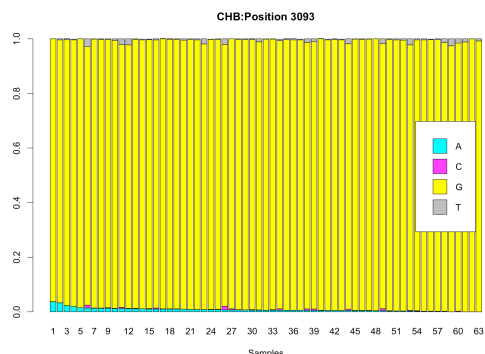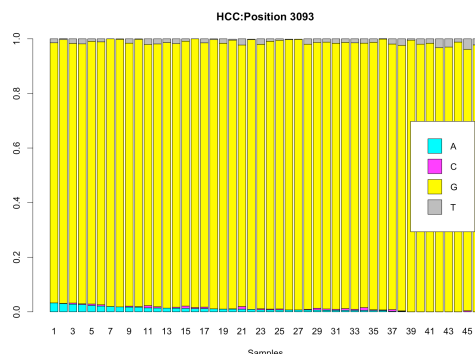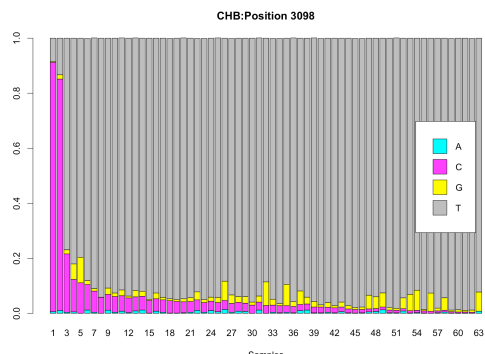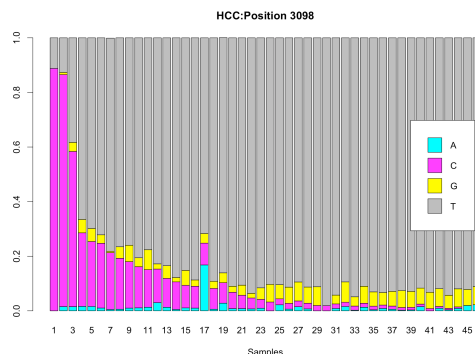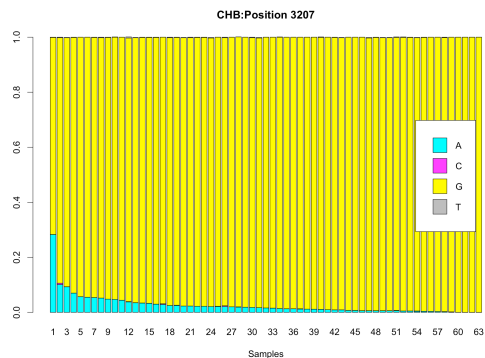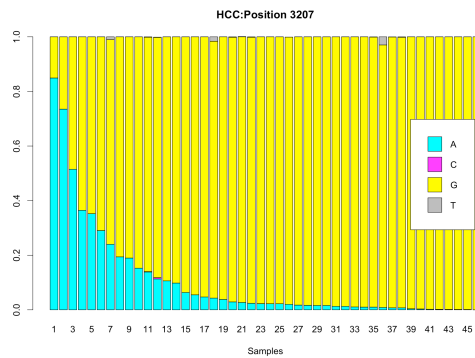

Supplement: Supplementary file 2 — Additional file 2. Figure S2: Nucleotide base frequency in HCC and CHB individuals of the ten point mutation positions selected by sparse logistic regression. [file 12985_2022_1836_MOESM2_ESM.pdf]

**A**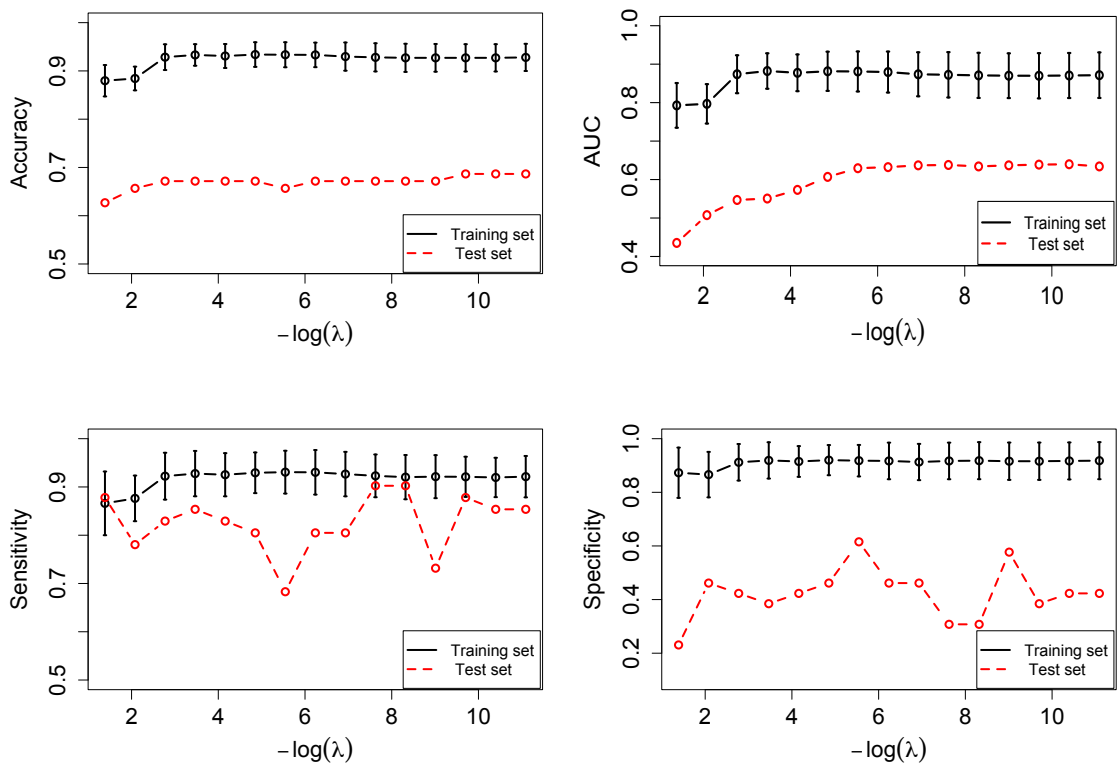**B**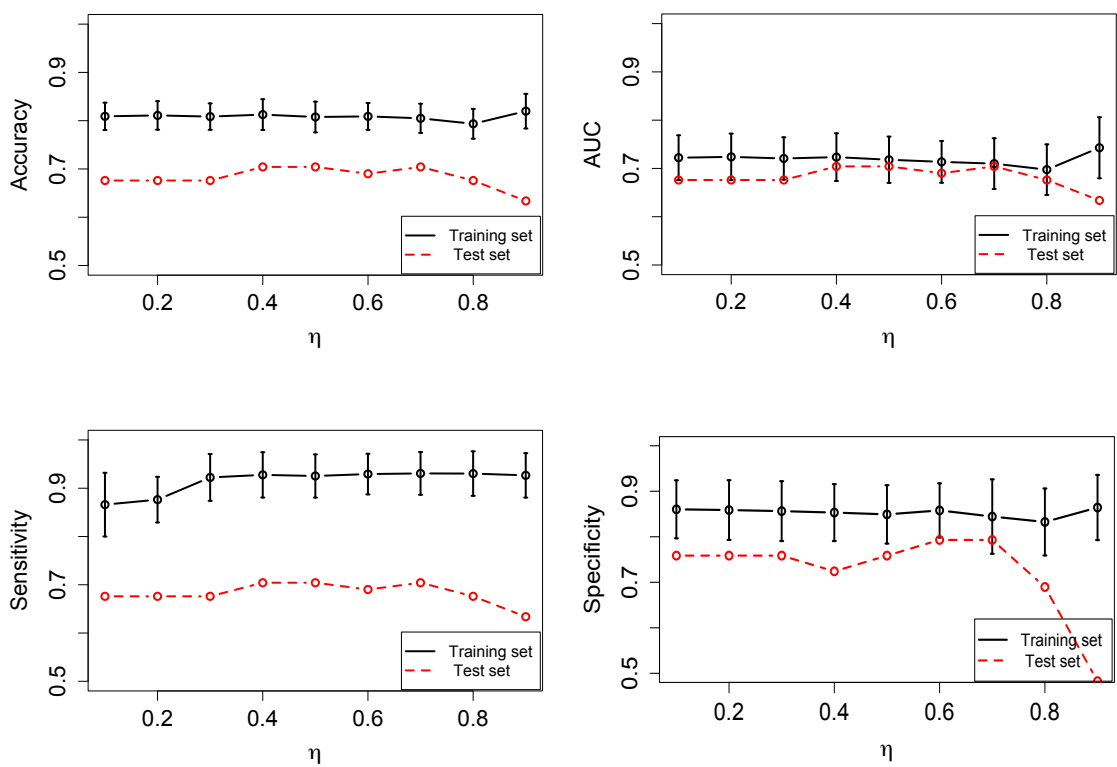

C

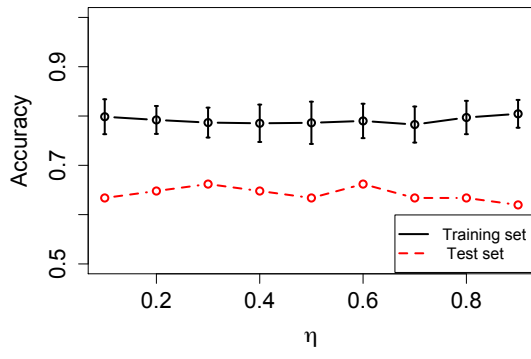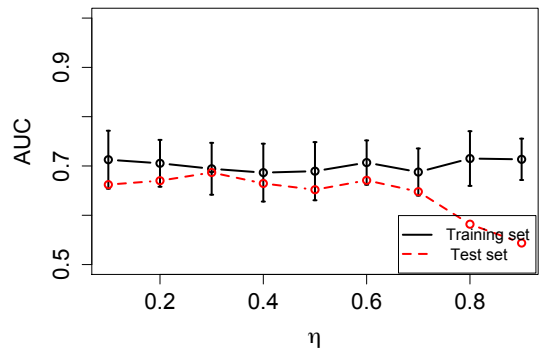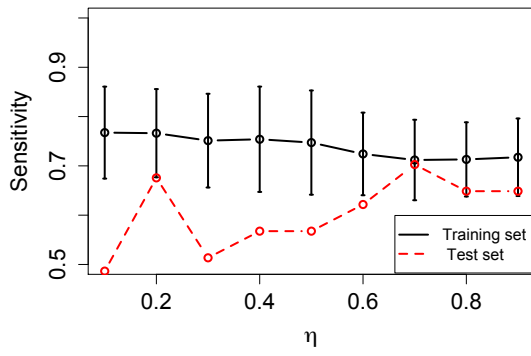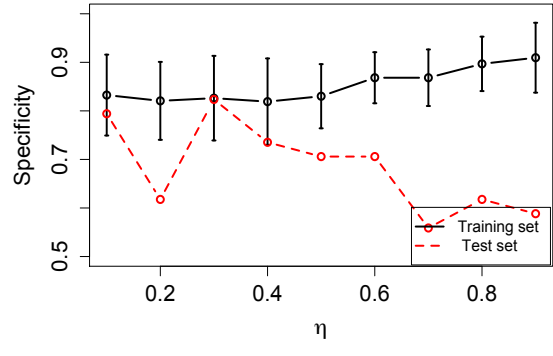

Supplement: Supplementary file 4 — Additional file 4. Figure S4: Association between three clinical variables and HBV quasispecies displayed by SLR with different parameters. A. Association between HBeAg and HBV quasispecies for different λs. B. Association between HBVDNA and HBV quasispecies for different ηs when K = 4. C. Association between ALP and HBV quasispecies for different ηs when K = 2. [file 12985_2022_1836_MOESM4_ESM.pdf]
