# Supplementary material for: Sparse logistic regression revealed the associations between HBV PreS quasispecies and hepatocellular carcinoma
Source: Virol J. 2022 Jun 28;19:114. doi: 10.1186/s12985-022-01836-9 (PMC9238101; doi:10.1186/s12985-022-01836-9)
Supplement: Supplementary file 3 — Additional file 3. Figure S3: Classification results for HCC/CHB patients using SLR in patients with different genotype of HBV.A Classification results for HCC/CHB patients of genotype B for different λs. B. Classification results for HCC/CHB patients of genotype C for different parameter λs. [file 12985_2022_1836_MOESM3_ESM.pdf]

Figure 2: A) Classification results for HCC/CHB patients of genotype B for different λs.
B) Classification results for HCC/CHB patients of genotype C for different parameter λs.

A

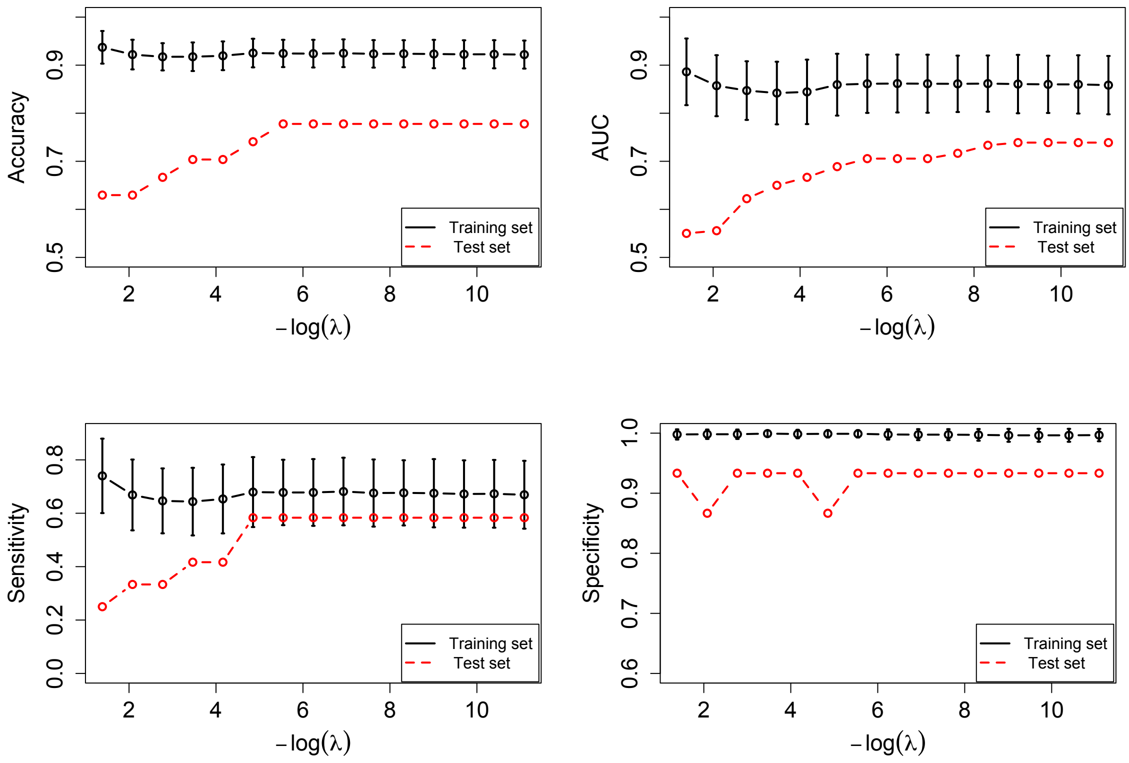

B

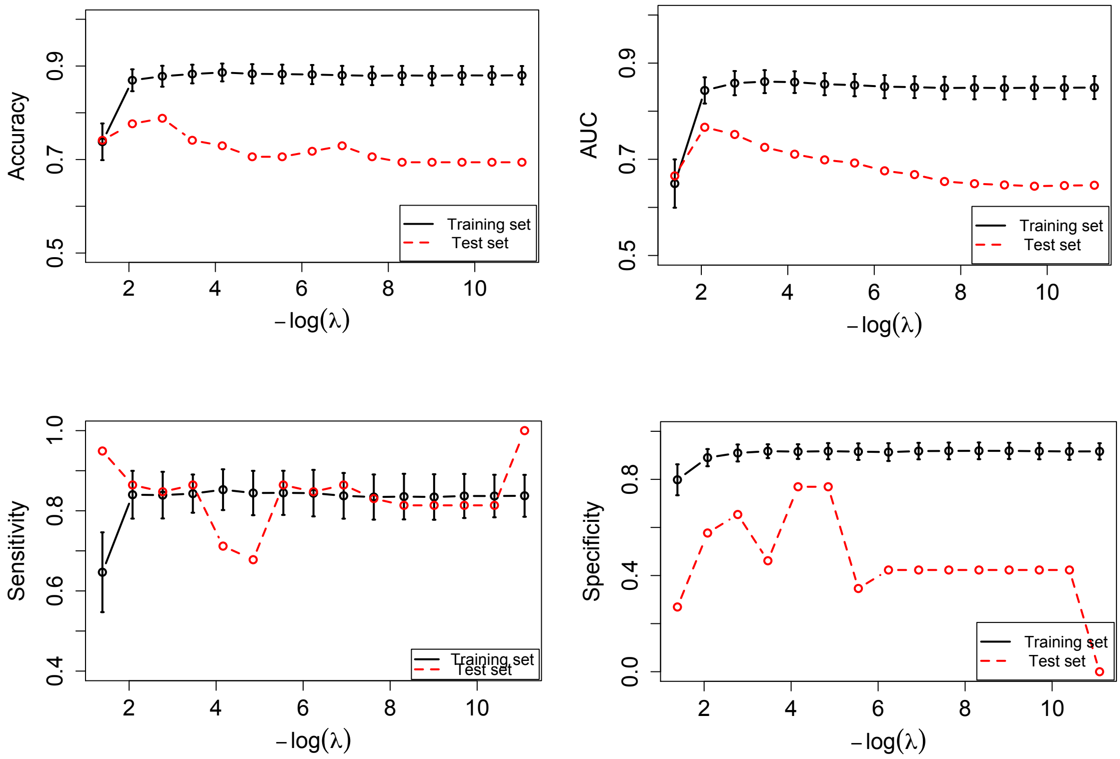

Accuracy
AUC
Sensitivity
Specificity
−log(λ)
Training set
Test set
