# Supplementary material for: Sparse logistic regression revealed the associations between HBV PreS quasispecies and hepatocellular carcinoma
Source: Virol J. 2022 Jun 28;19:114. doi: 10.1186/s12985-022-01836-9 (PMC9238101; doi:10.1186/s12985-022-01836-9)
Supplement: Supplementary file 6 — Additional file 6. Table S2: Classification results for HCC/CHB using LR, SLR, SVM and SSVM and calibrated SSVM. [file 12985_2022_1836_MOESM6_ESM.docx]

**Additional file 6**

**Table S2. Classification results for HCC/CHB using LR, SLR, SVM and SSVM and**

**calibrated SSVM.**

|  | LR | SLR (λ=0.5^3^) | | SVM | | | SSVM  (λ=0.5^5^) | Calibrated SSVM |
| --- | --- | --- | --- | --- | --- | --- | --- | --- |
|  | Test | Training | Test | | Test | Test | | Test |
| Accuracy | 0.688 | 0.861(0.032) | 0.794 | | 0.777 | 0.679 | | 0.696 |
| Sensitivity | 0.718 | 0.809(0.051) | 0.803 | | 0.775 | 0.662 | | 0.648 |
| Specificity | 0.634 | 0.898(0.019) | 0.780 | | 0.780 | 0.707 | | 0.780 |
| AUC | 0.644 | 0.883(0.043) | 0.795 | | 0.836 | 0.685 | | 0.722 |

LR: We applied the trained model in training set to the test set (Test set). SLR: We did cross validation within the training set (Training set), and applied the trained model to the test set (Test set). SVM: We applied the trained model in training set to the test set (Test set). SSVM: We did cross validation within the training set, and applied the trained model to the test set (Test set). Calibrated SSVM: Results using SSVM after model calibration using Platt.
