# Supplementary material for: Sparse logistic regression revealed the associations between HBV PreS quasispecies and hepatocellular carcinoma
Source: Virol J. 2022 Jun 28;19:114. doi: 10.1186/s12985-022-01836-9 (PMC9238101; doi:10.1186/s12985-022-01836-9)
Supplement: Supplementary file 7 — Additional file 7. Table S3: The selected positions associated with the three clinical parameters: HBeAg, HBVDNA, and ALP. [file 12985_2022_1836_MOESM7_ESM.docx]

**Additional file 7**

**Supplementary Table 3(Table S3). The selected positions associated with the three clinical parameters: HBeAg, HBVDNA, and ALP.**

| Characteristics | Selected positions |
| --- | --- |
| HBeAg | 19,26,36,2837,2857,2858,2860,2876,2898,2938,2959,2970 |
|  | 2996,3023,3033,3038,3090,3113,3176,3203,3211 |
| HBVDNA | 7,10,20,25,27,31,49,55,2857,2858,2889,2895,2898,2901 |
|  | 2910,2931,2934,2943,2946,2950,2980,2989,2997,3000,3009 |
|  | 3015,3026,3040,3063,3067,3088,3116,3117,3172,3206 |
| ALP | 10,23,25,27,40,45,49,2840,2860,2875,2889,2901,2910,2922 |
|  | 2931,2950,2951,2962,2964,2980,2988,2989,2996,3000,3003 |
|  | 3009,3015,3016,3021,3026,3057,3063,3086,3097,3102,3117 |
|  | 3120,3123,3126,3156,3169,3171,3186,3191,3208 |
